# Parliament and the Profession

**Published:** 1917-05-05

**Authors:** 


### Parliament and the Profession.

### Medical Arrangements in Macedonia.

The danger of further malarial sickness in Macedonia was the subject of a question by Sir H. Craik to the Under-Secretary of State for War. It was suggested that adequate medical precautions should be taken before the summer heat intervenes, unless the troops can be removed from the swampy ground near the river Struma. Mr. Macpherson assured the hon. member that every precaution would be taken to minimise the danger.

### Hospital for Paralysed Officers.

Sir H. Norman inquired whether a hospital in London, established by private beneficence for officers paralysed as the result of wounds, was now mainly used for ordinary cases, and was informed that the change had been made at the request of the hospital authorities, as only a limited number of beds had been required for paralysed officers.

### Venereal Disease—Hospital Cases.

In the course of the debate on the Venereal Disease Bill, Mr. Hayes Fisher stated that in London, in six hospitals out of the twenty-two which are dealing with these diseases, during the first three months of this year 1,195 males and 350 females, all new patients, were treated. In the Newcastle district 309 males and 154 females were treated in the same period.

### Cocaine Used in Dentistry.

It was stated that unregistered practitioners in dentistry had had their permission to use preparations containing not more than 1 per cent. of cocaine extended for a further period of three months from May 1.

### Medical Men Called Up.

Mr. Bonar Law, in answer to some questions, gave an assurance that the danger of the civil population in particular areas being left without doctors is fully realised, and although all the doctors of military age have been called up, they will only be taken after consultation, which is now taking place, with the Local Government Board and the National Health Commissioners.

### Venereal Disease—Compulsory Examination.

Commander Wedgwood asked the Home Secretary if women convicted of soliciting can now be medically examined for venereal disease if sent to prison, and he referred to the instance of two women sent to Holloway who had been so examined at the request of the magistrates. Mr. Brace said that all prisoners are medically examined on reception under the Statutory Prison Rules, but no prisoner is locally examined without his or her consent, and a magistrate has no power to order such an examination.

### Vaccine Making at St. Mary's Hospital.

The Financial Secretary to the War Office (Mr. Forster) informed Mr. Wing that grants-in-aid to cover the cost of materials used and the extra laboratory expenses involved in the preparation of vaccines for use in military hospitals, amounting to £4,465, have been made to the inoculation department of St. Mary's Hospital.

